# Metabolic Engineering of *Bacillus subtilis* for Riboflavin Production: A Review

**DOI:** 10.3390/microorganisms11010164

**Published:** 2023-01-08

**Authors:** Yang Liu, Quan Zhang, Xiaoxiao Qi, Huipeng Gao, Meng Wang, Hao Guan, Bo Yu

**Affiliations:** 1 Key Laboratory of Biofuels and Biochemical Engineering, SINOPEC (Dalian) Research Institute of Petroleum and Petro-Chemicals Co., Ltd., Dalian 116045, China; 2 CAS Key Laboratory of Microbial Physiological and Metabolic Engineering, State Key Laboratory of Mycology, Institute of Microbiology, Chinese Academy of Sciences, Beijing 100101, China; 3 School of Life Science, University of Chinese Academy of Sciences, Beijing 100049, China

**Keywords:** riboflavin, biosynthesis, *Bacillus subtilis*, metabolic engineering

## Abstract

Riboflavin (vitamin B_2_) is one of the essential vitamins that the human body needs to maintain normal metabolism. Its biosynthesis has become one of the successful models for gradual replacement of traditional chemical production routes. *B. subtilis* is characterized by its short fermentation time and high yield, which shows a huge competitive advantage in microbial fermentation for production of riboflavin. This review summarized the advancements of regulation on riboflavin production as well as the synthesis of two precursors of ribulose-5-phosphate riboflavin (Ru5P) and guanosine 5′-triphosphate (GTP) in *B. subtilis*. The different strategies to improve production of riboflavin by metabolic engineering were also reviewed.

## 1. Introduction

Riboflavin, also known as vitamin B_2_, is a water-soluble vitamin. Riboflavin does not have a direct metabolic function in living cells. It is primarily used as a derivative precursor for the in vivo synthesis of flavin nucleotides and flavin coenzymes, i.e., flavin mononucleotide (FMN) and flavin adenine dinucleotide (FAD), which act as coenzymes for all in vivo cellular redox reactions [1]. Thus, riboflavin is one of the key vitamins required to maintain normal metabolism. Riboflavin is endogenously biosynthesized in many plants and bacteria, whereas humans and animals must require dietary intake. Approximately 70% of commercially produced riboflavin is used as a nutritional additive in feed and 30% as a pharmaceutical and food additive [2,3]. Compared to chemical synthesis, microbial production has the advantages of low production cost, short production cycle, and environmental friendliness. Microbial synthesis of riboflavin has gradually replaced the traditional chemical synthesis. The most studied strains of in terms of metabolic pathways in the microbial fermentation for riboflavin production and regulation of gene expression are *Bacillus subtilis*, *Ashbya gossypii,* and *Corynebacterium ammoniagenes* [4]. Currently, the commercial production of riboflavin relies mainly on the fungus *A. gossypii* and the bacterium *B. subtilis*. Fungal production of riboflavin has the disadvantages of long fermentation cycle (6–7 d) and complex raw material composition ratios. In contrast, the bacterium *B. subtilis* has the advantages of short fermentation cycle, high yield, and simple raw material requirements. Therefore, *B. subtilis* has shown strong competitiveness in the microbial fermentation industry of riboflavin [5]. For example, the genetically engineered *B. subtilis* showed a maximum yield of 26.5 g/L of riboflavin after three days of fermentation [6]. The biosecurity and stable synthesis of the riboflavin precursors with high capacity, such as purine nucleoside, inosine and guanosine, is another fruitful advantage of *B. subtilis* [7]. Moreover, the physiological and genetic properties of *B. subtilis* have been well studied, hence most of the current research and applications of riboflavin are focused on *B. subtilis* strains. The *B. subtilis* based fermentative production of riboflavin has been industrialized for a long history in lots of companies, such as Hubei Guangji^®^ Pharmaceutical Co. Ltd., Wuhan, China as well as DSM^®^. In this paper, we summarize the recent progress of relevant research, with a focus on the work of summarizing regulatory mechanism of riboflavin biosynthesis and the genetic modification strategies for improving production in *B. subtilis*.

## 2. Riboflavin Biosynthesis Pathway in *B. subtilis*

**Figure 1:**
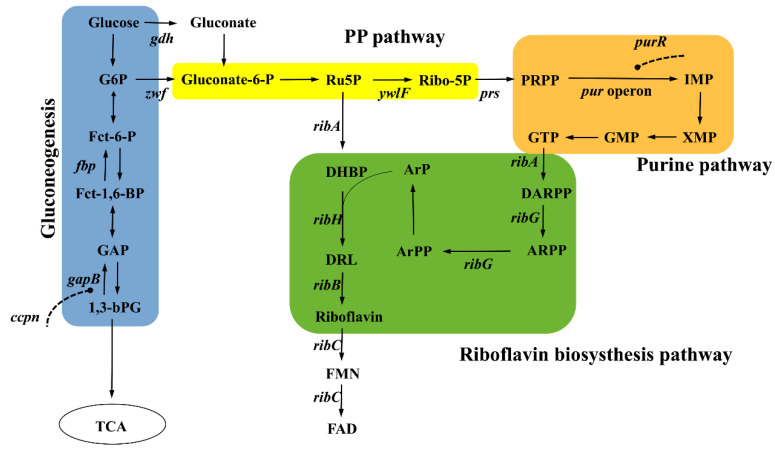
Biosynthesis and regulation of riboflavin production in *B. subtilis*.

As shown in Figure 1**,** the biosynthesis of riboflavin in *B. subtilis* involves both ribulose 5-phosphate (Ru5P) and guanosine 5′-triphosphate (GTP) as direct precursor substances that undergo a series of enzymatic reactions, which culminate in the production of the target product riboflavin.

G6P, glucose-6-phosphate; Fct-6-P, fructose-6-phosphate; Fct-1,6-BP, fructose-1,6-bisphosphate; GAP, glyceraldehyde-3-phosphate; 1,3-bPG, 1,3-bisphospho-glycerate; Ru5P, ribulose-5-phosphate; Ribo-5P, ribose-5-phosphate; PRPP, phosphoribosyl pyrophosphate; IMP inosine monophosphate; XMP xanthosine monophosphate; GMP guanosine monophosphate; GTP, guanosine-5′-triphosphate; DARPP, N-(2,5-Diamino-6-oxo-1, 6-dihydro-4-pyrimidinyl)-5-O-phosphono- β-D -ribofuranosylamine; ARPP, N-(5-Amino-2,6-dioxo-1,2,3,6-tetrahydro-4-pyrimidinyl)-5 -O-phosphono-β-D-ribofuranosylamin; ArPP, 1-[(5-Amino-2,6-dioxo-1,2,3,6- tetrahydro-4-pyrimidinyl) amino]-1-deoxy-5-O-phosphono-D-ribitol; ArP, 1-[(5-Amino-2,6-dioxo-1,2,3,6-tetrahydro-4-pyrimidinyl)amino]-1-deoxy-D-ribitol; DHBP, 2-Hydroxy-3-oxobutyl dihydrogen phosphate; DRL, 1-Deoxy-1-(6,7-dimethyl-2,4-dioxo-3,4-dihydro-8(2H)-pteridinyl)-D-ribitol; FMN, flavin mononucleotide; FAD, flavine-adenine dinucleotide; *fbp*:fructose-1,6-bisphosphatase 1; *gapB*, glyceraldehyde-3-phosphate dehydrogenase; *gdh*, glutamate dehydrogenase; *zwf*, glucose-6-phosphate dehydrogenase; *ywlf*, ribose-5-phosphate isomerase B; *prs*, PRPP synthetase;. ribA, GTP cyclohydrolase 2; ribB, 3,4-dihydroxy-2-butanone-4-phosphate synthase; ribC, riboflavin synthase; ribG, Diaminohydroxyphosphoribosylaminopyrimidine deaminase/5-amino-6-(5-phosphoribosylamino) uracil reductase; ribH, 6,7-dimethyl-8-ribityllumazine synthase. The dotted line represents negative feedback.

### 2.1. Upstream Synthetic Pathways—Precursors Ru5P and GTP Supply

In *B. subtilis*, Ru5P and GTP as precursors directly influence riboflavin production. The three main biosynthetic pathways of Ru5P in *B. subtilis* are the oxidative pentose phosphate pathway, non-oxidative pentose phosphate pathway and gluconate pathway. In the oxidative pentose phosphate pathway, glucose-6-phosphate is first converted to 6-phosphogluconolactone by glucose-6-phosphate dehydrogenase and then hydrolyzed to 6-phosphogluconic acid by 6-phosphogluconate dehydrogenase, which is further catalyzed to Ru5P through oxidative decarboxylation. In the non-oxidative pentose phosphate pathway, fructose-6-phosphate and glyceraldehyde-3-phosphate undergo transaldolase and transketolase reactions to produce Ru5P [8]. In the gluconate pathway, glucose is catalyzed by glucose dehydrogenase to gluconate, which is then phosphorylated by gluconate kinase to glucose-6-phosphate and enters into the oxidative pentose phosphate pathway to produce Ru5P [9].

GTP is formed in cells via the de novo purine synthesis pathway, which consists of 10 different enzymatic reactions to generate inosinemonophosphate (IMP) from 5-phosphoribosyl-1-pyrophosphate (PRPP) [10]. Next, IMP is converted to GTP and ATP. Genes involved in the purine pathway are clustered as one operon (*purEKBCSQLFMNHD*) in *B. subtilis* [11]. The transcription of all genes begins with a δ^A^ type promoter upstream of the gene *purE*, in which no internal promoter has yet been identified. In addition, purines can also be converted directly to their nucleoside monophosphate derivatives from intracellular PRPP via a purine salvage pathway [12].

### 2.2. Downstream Synthetic Pathways—Direct Riboflavin Biosynthesis

The riboflavin direct biosynthesis genes are present on chromosome in the form of operon. The total length of the operon is 4.2 kb and it contains five non-overlapping coding regions, in the order *ribG, ribB, ribA, ribH, ribT* (Table 1). Among them, *ribA* and *ribG* encode bifunctional enzymes. The *ribA* encodes GTP cyclohydrolase II at its 3′ end and hydroxybutyrone phosphate synthase at its 5′ end, which reacts with the precursors GTP and ribulose-5-phosphate, respectively. GTP cyclohydrolase II catalyzes the hydrolytic opening of the imidazole ring, releasing C-8 in the form of formate, and the hydrolytic release of pyrophosphate from the side chain of the ribose moiety [13] to produce 2,5-diamino-6-ribosylamino-4(3H)-pyrimidinone-5′-phosphate (DARPP), which requires Mg^2+^ for activation while inorganic phosphate acts as an inhibitor of the enzyme. Another function of *ribA* is to catalyze the production of L-3,4-dihydroxy-2-butanone-4-phosphate (DHBP) from Ru5P. RibA holoenzyme was found to be the rate-limiting enzyme in the riboflavin synthesis pathway. Overexpression of individual RibG, RibB, RibH and truncated RibA with GTP cyclohydrolase II or DHBP alone all decreased riboflavin production. While integration of intact RibA in the strain increased riboflavin production by 25% [14]. *ribG* encodes bifunctional pyrimidine deaminase and pyrimidine reductase activity [15], with a deaminase encoded at the 5′ end and a reductase at the 3′ end, which the enzyme is also activated by Mg^2+^. DARPP is deaminated in the second position of the pyrimidine ring in the presence of pyrimidine deaminase to produce 5-amino-6-ribosylamino-2,4(1H,3H)- pyrimidinedione-5-phosphate (ARPP). In the presence of pyrimidine reductase, NADPH is consumed to reduce and open the ring of the ribofuranose group of ARPP to form 5-amino-6-ribitylamino-2,4(1H,3H)-pyrimidinedione-5-phosphate (ArPP). This reduction reaction requires NADPH or NADH as a cofactor [16,17]. Subsequently, ArPP is catalyzed by a nonspecific phosphatase to dephosphorize into 5-amino-6-ribitylamino-2,4(1H,3H)-pyrimidinedione (ArP). However, the enzyme for this reaction has not yet been confirmed [13], and this dephosphorylation step is speculated to be catalyzed by a hydrolase with low substrate specificity [18].

Both *ribB* and *ribH* encode riboflavin synthases. Riboflavin synthase is a complex enzyme consisting of a light enzyme and a heavy enzyme. The light enzyme contains three alpha subunits. The heavy enzyme is a complex of one molecule of light enzyme and approximately 60 beta subunits [19]. *ribH* encodes the beta subunit of riboflavin synthase, also known as lumazine synthase. The reaction catalyzed by this enzyme is region-specific [20,21]. It forms a Schiff base by catalyzing the reaction between the 5-position amino group of ArP pyrimidine and the carbonyl group of C4 unit DHBP, followed by dephosphorylation and combination with a cross-tautomerization step to undergo ring closure to eventually produce 6,7-dimethyl-8-ribityllumazine (DRL). *ribB* encodes the alpha subunit of riboflavin synthase that catalyzes the dismutation of two molecules of DRL, the immediate precursor of riboflavin synthesis, which involves a C4 unit transfer [18], yielding one molecule of riboflavin and one molecule of ArPP. ArPP is recycled in the synthetic pathway as a substrate for lumazine synthase. *ribT is* located at the end of this operon and its function has not yet been elucidated [22]. *ribC* and *ribR* play an indirect regulatory role in riboflavin operon expression and their regulatory functions are described in detail later in this review.

## 3. Regulation on Riboflavin Synthesis

### 3.1. Upstream Pathway—Regulation of GTP Synthesis Module

Regulation of the riboflavin upstream synthesis pathway mainly includes transcriptional regulation of purine de novo synthesis pathway where the precursor GTP is located [23]. Expression of all coding genes that catalyze this anabolic pathway from PRPP to IMP (*purEKBCSQLFMNHD*) is subject to two types of regulation: transcriptional initiation regulation and transcriptional weakening regulation [24]. Special DNA sequences called Purboxes are present in the upstream control region of the purine operon transcription initiation site. PurR, the purine repressor, is encoded by *purR* and the effector is PRPP [25,26]. The PurR-PurBox system is involved in purine synthesis (*pur* operon), transport (*pbuG, pbuO*, and *pbuX*), metabolic function (*glyA* and *folD*), and other essential components of the transcriptional regulation of multiple genes [27]. When cells contain high concentrations of PRPP, PRPP binds to the repressor protein PurR, preventing PurR from binding to PurBox and thus allowing normal transcription of the purine operon. ADP is the major allosteric repressor of PRPP synthase, and its repressive effect is enhanced at elevated concentrations of ATP [28]. When cells contain higher concentrations of ADP, the reduced intracellular concentration of PRPP allows the inhibitor PurR to bind to PurBoxes, thereby inhibiting the transcriptional initiation of purine operons [29]. In addition to regulating purine pathway gene expression, PRPP competes with AMP for the catalytic binding site of PRPP amidotransferase encoded by *purF*, a key regulatory enzyme in the de novo synthesis of the purine pathway [30]. Thus, PRPP is an important signaling molecule for the synthesis of the riboflavin precursor GTP.

Transcriptional regulation of the de novo purine synthesis pathway is mediated by a switch in an RNA structure containing a G-box sequence that recognizes hypoxanthine and guanine [31]. With the inhibition of hypoxanthine and guanine, transcription of the purine operon is terminated before RNA polymerase enters the first structural gene of the operon [11,32]. When the cell contains higher concentration of guanine, guanine binds to the G-box, inducing the formation of a terminator structure in the mRNA leader region of the purine operon, the switch closes, and purine operon transcription is terminated prematurely. When the intracellular guanine concentration decreases, guanine dissociates from the G-box, an anti-terminator structure is formed in the purine operon mRNA leader region, the switch turns on, and purine operon transcription proceeds normally.

The purine pathway is tightly regulated by the two aforementioned mechanisms [30,33]. Therefore, to increase the anabolism of the precursor GTP, the purine operon regulatory mechanism needs to be partially deregulated. Early studies have shown that selection of the guanine structural analog 8-azaguanine (Az^r^) and the purine structural analog decoyinine (Dc^r^), methionine sulfoxide (MS^r^), psicofuranine and other resistant mutant strains can achieve the goal of deregulating the feedback of the GTP biosynthetic pathway and enhancing the metabolism of the purine pathway [34,35,36,37], the reaction mechanism of which is shown in Table 2. Ishii et al. selected *B. subtilis* with 8-nitroguanine resistance, which accumulated up to 18.0 g/L of guanosine and increased guanosine production by 80% compared to that of the starting strain [35]. Matsui et al. selected *B. subtilis* with DL-methionine sulfoxide resistance and obtained the strain AG169, which accumulated 8.0 g/L of guanosine and increased guanosine production by 45.5%, while accumulating 6.0 g/L of xanthine nucleoside [36]. Matsui et al. selected AG169 as the starting strain and selected psicofuranine resistant mutant strains GP-1, which accumulated 10.6 g/L of guanosine. Then, they used GP-1 as the starting strain to select resistance to Dc^r^ and obtained strain MG-1, which produced 16.0 g/L of guanosine [37]. For riboflavin synthesis, Perkins et al. introduced Az^r^, Dc^r^, and MS^r^ mutants. The acquisition of Az^r^ and Dc^r^ mutants was required to improve riboflavin yield by selecting for drug resistance strains, whereas MS^r^ did not have a significant effect on riboflavin yield improvement [1].

Recent studies have focused on genetically engineering targeted modifications of purine operon regulatory elements to increase gene expression levels of purine pathway. Asahara et al. knocked out the *purR* gene in *B. subtilis* 168, which increased the β-galactosidase activity encoded by the reporter gene *lacZ* by 5-fold. Deletion of the G-box in the purine operon mRNA leader region increased β-galactosidase activity by nearly 25-fold [38]. Lobanov et al. knocked out the *purR* gene in *B. subtilis* AM732 and deleted the G-box, overexpressed the purine operon gene and increased the copy numbers of *E. coli*-derived *purF* on the chromosome, resulting in a yield of 13.0 g/L of 5-aminoimidazole-4-carboxamide ribonucleoside [39,40]. However, it was not applied to riboflavin synthesis and thus, its effect on yield was unclear.

### 3.2. Downstream Pathways—Regulation of the Riboflavin Synthesis Module

Upstream of the *ribG* gene, the 5′ end of the *B. subtilis rib* operon contains a non-coding sequence of approximately 300 bp in length called *ribO*, which functions to regulate the transcription of riboflavin synthesis pathway genes. Mutation of the regulatory region *ribO* deregulates the repression of the riboflavin operons in *B. subtilis* and *B. amyloliquefaciens*. A potential transcriptional terminator has been observed between the translation initiation of *ribO* and the first gene of the riboflavin operon, *ribG* [41,42]. The regulation of this region involves a called ‘termination-anti-termination’ mechanism [41,43]. This sequence is conserved [41] and can fold into an RNA secondary structure with a basal stem and four hairpins, known as the RFN element [44]. Winker et al. showed that a specific sequence is present in the *ribO* region of the *rib* operon, which forms a stable hairpin structure upon transcription. This element could sense the concentration variations of FMN, which is the next intracellular metabolite of riboflavin, and can bind specifically to FMN, thereby regulating the transcription of the riboflavin synthetic gene operon [45].

*RibC* and *ribR* play an indirect role in the regulation of riboflavin operon expression. *ribC* encodes the bifunctional enzymes riboflavin kinase and FAD synthase, which sequentially catalyze the phosphorylation of riboflavin to generate FMN and then acetylated to generate FAD [46]. The N-terminal (amino acids 1–120) of RibR, the protein encoded by *ribR*, has a riboflavin kinase-like role and also catalyzes the production of FMN from riboflavin [47]. The C-terminus of RibR (amino acids 121–230) has no sequence similarity to other proteins with a known function [48]. In *B. subtilis*, *ribR is* generally silent in gene expression. Mack et al. showed that the expression of *rib* operon in *B. subtilis* is regulated by the small molecules FMN or FAD, but not by riboflavin [49]. Thus, although RibC and RibR are not directly involved in the synthesis process, they have an important role in maintaining intracellular riboflavin levels.

Excess FMN and FAD act as negative regulators of riboflavin production. Therefore, mutating the *ribC* gene to reduce the enzymatic activity of riboflavin kinase and FAD synthase can attenuate the negative feedback to the riboflavin production. Consequently, selection of resistant mutant strains of roseaflavin (the flavin analog) could obtain strains with better performance (Table 2) [50]. Previous studies have found that mutating the *ribC* gene leads to a reduction in riboflavin kinase activity and further reduces riboflavin conversion. Concurrently, mutation with reducing the intracellular FMN and FAD levels, relieving the transcriptional repression of the *rib* operon-related genes, increasing the transcriptional levels of ribP_1_ and ribP_2_, as well as enabling the sustained expression of genes in the riboflavin biosynthetic pathway could increase the accumulation of riboflavin [1,10,49,50].

## 4. Metabolic Engineering Strategies to Increase Riboflavin Production

Under natural conditions, *B. subtilis* does not accumulate riboflavin. To enable the overproduction of riboflavin, the construction strategy should include two main aspects: (1) deregulation of the riboflavin biosynthetic pathway and (2) enhancing expression of riboflavin operon-related genes. Stepanov et al. constructed a riboflavin-producing strain *B. subtilis* 304/pMX45, which possessed 8-azaguanine and roseaflavin resistance markers, by relieving feedback inhibition in the synthesis pathway. The riboflavin synthesis gene operon was overexpressed in plasmid pMX45. The recombinant strains produced 1.6 g/L riboflavin after 40 h fermentation, and a higher titer of 4.5 g/L was achieved by further optimization of the medium and fermentation process. Microbial metabolic engineering is being widely applied to the modification of *B. subtilis* for riboflavin overproduction. As summarized in Table 3, the riboflavin synthesis level in the engineered strains is significantly improved. The strategies of strain engineering were reviewed in detail as follows.

### 4.1. Engineering Upstream Synthetic Pathway

#### 4.1.1. Enhancing Pentose Phosphate Pathway for Ribulose-5-Phosphate Supply

The precursor substance Ru5P is mainly derived from the pentose phosphate pathway. The glucose-6-phosphate dehydrogenase that is encoded by *zwf* is one of the key enzymes in the oxidative branch of the pentose phosphate pathway (PP), whose role is to convert glucose-6-phosphate to 6-phosphogluconate (Figure 1), accompanied by the reduction of NADP^+^ to NADPH. Subsequently, 6-phosphogluconate is oxidized to Ru5P, the enzyme that has a more direct impact on riboflavin production [10,51]. Duan et al. integrated the xylose-inducible promoter P_xyl_ into the chromosomal *zwf* locus, increased the metabolic flux of the PP pathway and concentration of the precursor Ru5P in cells nearly 4-fold [52]. In addition to overexpressing gene *zwf* in the PP pathway, Zhu et al. overexpressed glucose dehydrogenase encoded by *gdh* of the gluconate bypass pathway with strong promoter P_43_, which improved cell growth and riboflavin synthesis [53]. Zhang et al. overexpressed *gntP*, which encodes gluconate permease, to increase riboflavin production [54]. By constructing the tunable intergenic regions library, the gene expression of *zwf*, *ribBA* and *ywlF* were adjusted to increase the intracellular Ru5P and produced 2.7 g/L riboflavin in shaking flask, which increased the yield by 64.35% [55].

Glucose-6-phosphate dehydrogenase (G6PD) and 6-phosphate dehydrogenase (6PGD, encoded by gene *gnd*) are inhibited by the allosteric effect of intracellular metabolites. It has been found that mutant enzymes G6PD^A243T^ and 6PGD^S361F^ of *Corynebacterium glutamicum* can weaken the negative feedback inhibition. Thus, introducing the two mutant enzymes G6PD^A243T^ and 6PGD^S361F^ of *C. glutamicum* with high affinity for substrates glucose-6-phosphate and 6-phosphogluconate in the strain increased the precursor Ru5P and then promoted riboflavin production by 17% [56]. Tannler et al. increased the intracellular accumulation of the precursor Ru5P by knocking out the inhibitor, encoded by *ccpN*, to release the repressed expression of the gluconeogenic genes, *gapB* and *pckA*, which improved the riboflavin production by 63% [57]. Furthermore, saving Ru5P for riboflavin production is another effective strategy. Inactivation of ribulose-5-phosphate-3-epimerase (Rpe) could reduce Ru5P depletion and then increase carbon flux of riboflavin biosynthesis. In this case, the riboflavin production increased more than 5-fold compared with that of the parental strain [58]. In addition, reducing power NADPH is also the crucial factor for riboflavin biosynthesis. To balance NADPH in vivo, the gluconeogenesis pathway was regulated by overexpression of glyceraldehyde-3-phosphate dehydrogenase to increase the metabolic flux of oxidative pentose phosphate pathway, the major route for NADPH generation, which resulted in a 27% increase in riboflavin yield [59].

#### 4.1.2. Enhancing De Novo Purine Synthesis Pathway

It has been shown that the addition of GTP can increase the production of riboflavin [60,61], hence an efficient supply of purine is essential [62,63,64,65]. Shi et al. regulated the de novo purine synthesis pathway to increase riboflavin production. Using transcriptomic analysis, the genes related to the purine de novo synthesis were found to be downregulated in the riboflavin accumulating strain *B. subtilis* RH33, compared to the wild strain *B. subtilis* 168. Additionally, overexpression of phosphoribosyl pyrophosphate synthase and ribose-5-phosphate isomerase resulted in a 4.7-fold increase in PRPP content and a 25% increase in riboflavin production [66]. GTP is mainly produced via the de novo purine biosynthetic pathway and it can also be synthesized from purine bases or purine nucleosides via the salvage pathway. These reactions are catalyzed by purine phosphoribosyltransferases. Knockout of adenine phosphoribosyltransferase (*apt*), xanthine phosphoribosyltransferase (*xpt*) and adenine deaminase (*adeC)* increased riboflavin production by 14.02%, 6.78%, and 41.50%, respectively [67]. Disruption of the negative regulator *purR* resulted in 380-fold upregulation of transcription levels of related purine synthesis genes. Meanwhile, overexpression of the site mutated *purF*, which reduced feedback repression, improved PRPP amidotransferase enzyme activity, and thus increased purine pathway metabolic flux [24]. The purine metabolic pathway is tightly regulated. Although overexpression of *purF* alone did not significantly increase the yield of riboflavin, co-expression of *purFMNHD* effectively increased the amount of GTP [24].

### 4.2. Engineering the Downstream Synthetic Pathway—Riboflavin Synthesis Module

Perkins et al. constructed a series of strains, derived from strain *B. subtilis* 168, by integrating multiple copies of the riboflavin operons into the chromosome. Additionally, the two natural promoters ribP_1_ and ribP_2_ on the riboflavin operon were replaced with the strong constitutive promoter P_15_ of phage SPO1 to further improve riboflavin production [5]. The relationship between operon dose and yield was investigated using multiple tandem amplification of riboflavin operons on chromosomes. The study showed that each increase in riboflavin operon dose within a specific range increased the yield by approximately 0.4 g/L [68]. On the basis of increasing gene operon copies in chromosome, Van et al. inserted the strong VegI promoter in the chromosome that drives the expression of *ribA* gene, and the yield increased by approximately 25% [69]. Lehmann et al. mutated *ribA* by multiple rounds of error-prone PCR and obtained a mutant Construct E, which led to a 2-fold increase in enzyme activity and a 4-fold increase in *K_m_* value in *E. coli* [70]. However, the effect of this mutation on riboflavin production in *B. subtilis* was not mentioned.

### 4.3. Enhancement of Energy Supply to Increase Riboflavin Production

In addition to modifications related to the direct synthetic pathway, microbial energy metabolism is another essential factor, which make contributions to relieve the limits of yield increases of many bio-products [7,71]. ATP is vital for synthesis of PRPP and involved in several steps of riboflavin biosynthesis. Thus, increasing energy supply should be an effective way to increase riboflavin production in *B. subtilis* [72,73]. Sauer et al. investigated the basal metabolic capacity of *B. subtilis* by flux balance model and found that cellular energy is the limiting factor for riboflavin production. Calculation results showed that an increase in the P/O ratio from 1.3 to 1.5 during oxidative phosphorylation in *B. subtilis* could increase riboflavin synthesis capacity by 20% [7]. In *B. subtilis*, aerobic respiration is the main mode of cellular energy production and the respiratory chain of *B. subtilis* can pump up to 4 protons per electron transfer. Its respiratory chain consists of the following two main branches: the quinone oxidase branch and the cytochrome oxidase branch [74]. The quinone oxidases include cytochrome aa_3_ (H^+^/e− = 2) and cytochrome bd (H^+^/e^−^ = 1), and the cytochrome oxidases include cytochrome caa_3_. A previous study demonstrated that the cytochrome caa_3_ pathway is negligible in *B. subtilis*, and that aerobic growth of the bacterium requires either cytochrome aa_3_ or cytochrome bd [75]. Zamboni et al. knocked out the gene encoding cytochrome aa_3_, which resulted in a drastic reduction in the TCA cycle metabolic flux and led to an increase in by-products, whereas knocking out the gene encoding cytochrome aa_3_ and cytochrome bd led to weak growth in the strain under aerobic conditions [75]. Li et al. improved riboflavin production by knocking out the gene encoding cytochrome bd oxidase, which allowed electron flow into the high-coupling-efficiency aa_3_ pathway in the respiratory chain [76]. Duan et al. expressed the hemoglobin gene *vgb* of *Vitreoscilla* in *B. subtilis*, which improved oxygen transport and resulted in an increase in riboflavin production by approximately 20% [77]. Moreover, energy supply is closely related to the TCA cycle. Regulating the efficiency of energy supply can adjust glucose consumption and the TCA cycle to a suitable ratio, thereby alleviating overflow metabolism and reducing the accumulation of the acetate as a by-product. Due to the lack of the glyoxylate shunt pathway in *B. subtilis*, acetoin was catabolized in the TCA cycle which increased the intracellular ATP to ADP ratio by 5.8-fold. This affects the intracellular energy metabolism, which increased the intracellular GMP to GTP metabolic flux and thus improved riboflavin production [78].

**Table 3:** Metabolic engineering of *Bacillus subtilis* for riboflavin overproduction.

Target Gene	Method	Strain Background	VB_2_ Improvement ^a^	VB_2_ Titers or Yields ^b^	Reference
*ribA*	VegI promoter	*B. subtilis* RB50::[pRF69]n[pRF93]m Ade^+^	1.25	17.5 g/L	[69]
*rib* operon	Multiple copies, VegI promoter	*B. subtilis* RB9	280	14 g/L (0.02–0.05 g/L)	[5]
*zwf*	P_xyl_ promoter	*B. subtilis* RH33	1.25	0.05 g/g Glc (0.04 g/g Glc)	[52]
*zwf*	Site-directed mutagenesis	*B. subtilis* RH33	1.11	0.052 mmol/g CDW/h (0.047 mmol/g CDW/h)	[56]
*zwf*	Double site-directed mutagenesis with *gnd*	*B. subtilis* RH33	1.17	0.055 mmol/g CDW/h (0.047 mmol/g CDW/h)	[56]
*gdh*	P_43_ promoter	*B. subtilis* RH33::[pRB63]_n_	1.60	0.047 g/g CDW (0.03 g/g CDW)	[53]
*gapB, fbp*	P_43_ promoter	*B. subtilis* RH33	1.27	13.36 g/L (10.5 g/L)	[58]
*ccpn*	Deletion	*B. subtilis* RB50::pRF69	1.63	0.062 g/g Glc (0.038 g/g glc)	[59]
*prs, ywlF*	P_43_ promoter	*B. subtilis* RH33	1.25	15 g/L (12 g/L)	[61]
*purR, purF*	*purR* deletion; *purF* overexpression	*B. subtilis* 168	3	826 mg/L (275 mg/L)	[24]
*purFMNHD*	P_43_ promoter	*B. subtilis* RH33	1.24	0.031 g/g Glc (0.025 g/g Glc)	[24]
*cyd*	Deletion	*B. subtilis* RH50::[pRB69]_n_	1.38	12.3 g/L (8.9 g/L)	[75]
*cyd*	Deletion	*B. subtilis* PK	1.4	0.07 mmol/g CDW/h (0.05 mmol/g CDW/h)	[76]
*pta, alsS*	*pta* deletion *alsS* overexpression	*B. subtilis* RH33::[pRB63]_n_	1.5	0.045 g/g CDW (0.03 g/g CDW)	[78]

^a^ The unit for VB_2_ improvement is fold increase. **^b^** The titers or yields in parenthesis indicated the data from the starting strains.

In summary, early studies on engineering riboflavin production in *B. subtilis* focused on the direct pathway of riboflavin synthesis, including enhanced expression of related functional genes by changing strong promoters, increasing overall operon gene dose, or debugging the rate-limiting enzyme RibA by gene overexpression, all of which are useful to increase riboflavin production in *B. subtilis* to some extents [5,69,70]. More recent studies on engineering riboflavin synthesis have mainly focused on the rate-limiting enzyme gene *ribA*. However, balancing the expression of the five genes in the metabolic pathway, such as regulating the RBS intensity of each gene separately at the pathway level to achieve optimal regulation of riboflavin production, has yet to be reported. Overexpression of exogenous genes or mutants with higher enzymatic activity to further enhance riboflavin production is also possible. Although the downstream pathway of riboflavin synthesis has a more direct impact on improving riboflavin production, the enhancement is still limited. To further enhance riboflavin production, more carbon fluxes need to be introduced in the direction of riboflavin synthesis to enhance precursor supply. Thus, the focus shifted to the precursor supply and energy metabolism during riboflavin synthesis, which has an important effect on riboflavin production. Furthermore, the riboflavin synthesis requires lots of energy. Therefore, improving the intracellular ATP level can effectively enhance riboflavin synthesis capacity and yield in *B. subtilis*. Given the energy metabolism, the possibility of using other carbon sources to enhance the carbon flow to the riboflavin synthesis pathway and increase the intracellular ATP content should also be considered. In addition, several fundamental issues in the riboflavin biosynthetic pathway remain to be addressed, which hindered the strain engineering: (1) the specific function of the riboflavin operon *ribT*. Although Yakimov et al. predicted the distribution of the secondary structure of the RibT protein and proposed a tertiary structure for RibT, the catalytic function of the enzyme is still unknown [22]; (2) the specific mechanism of the dephosphorylation reaction in the riboflavin terminal synthesis pathway needs to be elucidated; and (3) the specific mechanism and regulation of riboflavin secretion in *B. subtilis* is not clear. Studies have shown that riboflavin transport systems exist in many microorganisms. The ability of many microorganisms to accumulate riboflavin in the culture medium suggests that riboflavin can be efficiently secreted by the organisms [1]. Riboflavin transport in *B. subtilis* occurs via a specific carrier-mediated process with very high affinity (K_m_ values between 5 and 20 nM). Experimental evidence has been presented that the gene *ypaA* might encode a riboflavin transporter in *B. subtilis* [79] and that exogenous intake of riboflavin inhibits the synthesis of the protein [80]. However, studies on bacterial riboflavin transport are currently limited to uptake from the medium and little is known about the mechanism of riboflavin efflux in bacteria. Hemberger et al. overexpressed the riboflavin transporter protein RibM from *Streptomyces subtilis* in *B. subtilis* for catalytic riboflavin secretion. However, the study was not fully confirmed whether RibM enhances riboflavin efflux [81]. The abovementioned issues need to be clarified to identify important targets to further improve the efficiency of riboflavin synthesis.

## 5. Concluding Remarks and Perspectives

Industrial biosynthesis of riboflavin is one of the successful projects in the field of biotechnology and metabolic engineering. Within a short period, fermentative production has managed to completely replace chemical synthesis, which has a history of more than 50 years. The interest in the riboflavin market is growing with an increasing demand for nutrition. Although industrial bio-production of riboflavin is well documented, there is still considerable scope for improvement. The chemometric models predict a maximum theoretical conversion rate of 0.16 mol riboflavin/mol glucose for the synthesis of riboflavin in *B. subtilis* [7]. Considerable research on strain breeding and genetic modification has been conducted, while the current yield of riboflavin from glucose is still far below the theoretical value. To improve the riboflavin conversion rate, more carbon flux needs to be directed to riboflavin synthesis. Given the research experience, single-gene knockout and gene overexpression are normally not sufficient yet. As the model Gram-positive bacterial strain, *B. subtilis* have been widely investigated [82]. A variety of genetic manipulation tools and strategies targeting gene expression regulation have been successfully applied in *B. subtilis*, such as the CRISPRi system [83], CRISPR-Cas9 genome editing system [84], an expression system based on an engineered promoter [85] and an expression system based on an endogenous type II toxins and antitoxins [86]. T‘he development of gene manipulation has facilitated the in-deep study of metabolic pathway regulatory mechanisms and metabolic engineering [87]. To date, the genome-scale metabolic model of *B. subtilis* has been well established and is continuously being updated [88,89,90]. Thus, the application of the above-mentioned techniques will rapidly promote the strain engineering of riboflavin production. Additionally, the problem of multiple regulatory mechanisms impeding the redistribution of metabolic fluxes is ignored in current metabolic engineering [87]. The introduction of a more systematic approach is necessary to improve yields and titers. The rapid advances in systems biology enable the analysis of strain metabolism via multiple layers of omics, including transcriptome, proteome, metabolome and promoter activity, using statistical and computational models [91,92]. Based on the multifaceted omics, various new targets for the modification of riboflavin strains could be demonstrated on a system level [93]. For example, a new mutant locus of two-component response regulator YvrH^R222Q^ that deregulates the purine de novo synthesis pathway to improve riboflavin production was recently identified [94]. Hypoxia was found to affect purine and nitrogen metabolism by transcriptomic analyses. Thus, the riboflavin synthesis was enhanced by dynamically regulating the exogenously introduced *vgb* gene expression to improve oxygen utilization [95]. Furthermore, post-translational engineering, allosteric engineering to release inhibition on key enzymes and dynamic control pathway flux will also help regulate the engineered *B. subtilis* to achieve the desired cellular traits [87]. Finally, some riboflavin-producing strains currently used in industry are engineered strains with antibiotic gene markers, which could not be allowed for some purposes such as in the European food industry [96,97,98]. Therefore, there is an urgent need to develop more efficient and food-safe strains for riboflavin production. The use of new genetic manipulation tools, such as CRISPR-based genome editing, to construct a new generation of highly efficient and food-safe riboflavin-producing strain, is expected. Recently, one rationally engineered *E. coli* strain with high capacity of production of riboflavin was constructed. The riboflavin titer reached 21 g/L in fed-batch fermentation with a yield of 0.11 g riboflavin/g glucose [99]. Thus, in addition to the *B. subtilis* host, other hosts with clear genetic background and easy manipulation features, like *E. coli* host, will be another choice for highly efficient production of riboflavin in this rapidly developing era of synthetic biology.

## Figures and Tables

**Table 1:** Riboflavin biosynthetic and regulatory genes in *B. subtilis*.

Gene	Function	Optimum pH *	Optimum Temperature *	K_m_ Value (μM) *	Cofactor	EC Number
*ribG*	Diaminohydroxyphosphoribosylaminopyrimidine deaminase/5-amino-6-(5-phosphoribosylamino) uracil reductase	8.0	37	-/0.005	-/ NADPH	EC:3.5.4.26/ 1.1.1.93
*ribB*	riboflavin synthase	7.4	37	0.010–0.030	-	EC:2.5.1.9
*ribA*	GTP cyclohydrolase II/3,4-dihydroxy-2-butanone-4-phosphate synthase	8.5/8.0	37	0.031–0.112/ 0.116–0.181	-	EC:3.5.4.25/ 4.1.99.12
*ribH*	6,7-dimethyl-8-ribityllumazine synthase	7.0	37	0.130	-	EC:2.5.1.78
*ribT*	unknown	-	-	-	-	-
*ribC*	riboflavin kinase/FAD synthetase	8.5/-	52/-	0.180/-	ATP	EC:2.7.1.26/ 2.7.7.2
*ribR*	riboflavin kinase	8.5	52	0.180	ATP	EC:2.7.1.26

* Data from Brenda enzyme database.

**Table 2:** Mechanism and effect of reagents used for screening riboflavin-overproducing strains.

Screening Drugs	Analogue	Reaction Mechanism	Strain Background	Guanosine Improvement	Reference
8-azaguanine	Guanine	Deregulation of the feedback of PRPP amidotransferase	RDA-16	1.6–1.8	[35]
Methionine sulfoxide	Purine	Enzyme activity of 5′ -nuclease decreased and enhance XMP synthesis from IMP	AG169	1.455	[36]
Psicofuranine	Purine	Improve enzyme activity of GMP synthetase	GP-1	1.325	[37]
Decoyinine	Purine	Enhance GMP synthesis from XMP	MG-1	1.509	[37]

## Data Availability

Not applicable.
